# Chinese Healthcare Professionals’ Perspectives on the Application of AI in Elderly Care

**DOI:** 10.1093/geroni/igaf122.2823

**Published:** 2025-12-31

**Authors:** Xiuhong Zhang, Jinxia Lu

**Affiliations:** Shanxi Bethune Hospital, Taiyuan, Shanxi, China (People’s Republic); Shanxi Bethune Hospital, Taiyuan, Shanxi, China (People’s Republic)

## Abstract

Background Ageing populations, growing burden of chronic diseases, and rising costs of healthcare worldwide are posing unprecedented challenges for governments. While artificial intelligence (AI) shows considerable promise in enhancing clinical decision-making processes and optimizing healthcare efficiency, its successful implementation depends on addressing healthcare professionals’ acceptance, concerns, and preferences. This study investigates health professionals’ perspectives on the perceived benefits, potential risks, and implementation strategies for AI adoption in elderly care. Methodology A qualitative, multi-center study was conducted across five cities China. The study included 55 healthcare professionals—physicians, nurses, and allied health practitioners—who participated in 11 focus group discussions. Participants were recruited through purposive and snowball sampling. Semi-structured interviews explored their attitudes toward AI in elderly care, and thematic analysis was used to identify key themes. Results Participants recognized AI’s potential to improve efficiency, support clinical decision-making, and enhance patient care. Many highlighted its role in reducing workload and aiding early disease detection. However, concerns about data security, ethical implications, and loss of human-centered care were common. Challenges related to trust in AI, workflow integration, and training needs were also emphasized. Preferred implementation strategies included clear regulations, human-AI collaboration, and institutional support for skill development. Conclusion Healthcare professionals view AI as a valuable tool but also state the need for ethical oversight and proper training. This study provides insights into the Chinese healthcare professionals’ views and preferences to inform the delivery of an AI in elderly care in the Chinese context.

